# Seasonal Variations of Solar‐Induced Fluorescence, Precipitation, and Carbon Dioxide Over the Amazon

**DOI:** 10.1029/2021EA002078

**Published:** 2022-01-18

**Authors:** Ronald Albright, Abigail Corbett, Xun Jiang, Ellen Creecy, Sally Newman, King‐Fai Li, Mao‐Chang Liang, Yuk L. Yung

**Affiliations:** ^1^ Department of Earth & Atmospheric Sciences University of Houston Houston TX USA; ^2^ SeekOps Inc Austin TX USA; ^3^ Bay Area Air Quality Management District San Francisco CA USA; ^4^ Department of Environmental Sciences University of California Riverside CA USA; ^5^ Institute of Earth Sciences, Academia Sinica Taipei Taiwan; ^6^ Division of Geological and Planetary Sciences, California Institute of Technology Pasadena CA USA; ^7^ Jet Propulsion Laboratory Pasadena CA USA

**Keywords:** Photosynthesis, burned area, vapor pressure deficit, biosphere‐atmosphere exchange

## Abstract

Previous studies suggested that the Amazon, the largest rainforest on Earth, changes from a CO_2_ sink to a CO_2_ source during the dry/fire season. However, the biospheric contributions to atmospheric CO_2_ are not well understood during the two main seasons, the dry/fire season and the wet season. In this article, we utilize Orbiting Carbon Observatory 2 (OCO‐2) Solar‐Induced Fluorescence (SIF) to explore photosynthetic activity during the different seasons. The spatiotemporal variability of OCO‐2 SIF, OCO‐2 CO_2_, precipitation, and burned area are investigated over the Amazon from September 2014 to December 2019. Averaging over the entire Amazon region, we found a positive temporal correlation (0.94) between OCO‐2 SIF and Global Precipitation Climatology Project precipitation and a negative temporal correlation (−0.64) between OCO‐2 SIF and OCO‐2 CO_2_, consistent with the fact that precipitation enhances photosynthesis, which results in higher values for SIF and rate of removal of CO_2_ from the atmosphere above the Amazon region. We also observed seasonality in the spatial variability of these variables within the Amazon region. During the dry/fire (August–October) season, low SIF values, low precipitation, high vapor pressure deficit (VPD), large burned areas, and high atmospheric CO_2_ are mainly found over the southern Amazon region. In contrast, during the wet season (January–March), high SIF values, high precipitation, low VPD, smaller burned areas, and low CO_2_ are found over both the central and southern Amazon regions. The seasonal difference in SIF suggests that photosynthetic activity is reduced during the dry/fire season relative to the wet season as a result of low precipitation and high VPD, especially over the southern Amazon region, which will contribute to more CO_2_ in the atmosphere during the dry/fire season.

## Key Points

- The anomalously high SIF region moves with the precipitation from the north to the south of the Amazon from the dry/fire season to the wet season
- Photosynthetic activity is reduced over the southern Amazon region during the dry/fire season as a result of low precipitation and high vapor pressure deficit
- Seasonal differences in SIF suggest that less CO_2_ will be removed from the atmosphere through photosynthesis during the dry/fire season

## 1 Introduction

Under favorable conditions such as sufficient nutrients and soil water, plants utilize sunlight, carbon dioxide (CO_2_), and water to produce glucose by photosynthesis. Thus, photosynthesis removes CO_2_ from the atmosphere, acting as a carbon sink (Pearman & Hyson, 1980, 1981). In addition to the release of oxygen molecules as a byproduct during photosynthesis, chlorophyll also emits light in the red and near‐infrared wavelength range, known as solar‐induced fluorescence (SIF), which has been shown to be measurable from space (Baker, 2008; Frankenberg et al., 2011; Joiner et al., 2012, 2011; Papageorgiou & Govindjee, 2004). SIF products derived from satellite data have been used to assess photosynthetic activity of the biosphere (Frankenberg et al., 2011; Raychaudhuri, 2014) and track atmospheric oxygen‐carbon‐dioxide balance (Raychaudhuri, 2014). SIF data have also been utilized to explore the carbon balance during different seasons in Amazonia (Lee et al., 2018; Parazoo et al., 2013).

Estimating SIF through remote sensing techniques is challenging due to its weak signal. Current space‐based measurements that have enough information content to retrieve SIF include the Medium Resolution Imaging Spectrometer sensor on the Environmental Satellite Platform (Guanter et al., 2007), the Fourier Transform Spectrometer sensor on the Greenhouse Gases Observing Satellite (GOSAT) platform (Guanter et al., 2012; Joiner et al., 2011), the UV/visible cross‐track scanning spectrometer on the Global Ozone Monitoring Instrument 2 (GOME‐2) (Joiner et al., 2013), and the Orbiting Carbon Observatory 2 (OCO‐2) (Frankenberg et al., 2014; Sun et al., 2017). SIF has been estimated using the oxygen A‐band from the GOSAT satellite (Hamazak et al., 2005; Kuze et al., 2009). Joiner et al. (2013) showed that SIF can also be derived using GOME‐2 spectra at the 866 nm or at 715–780 nm wavelengths, despite their moderate spectral resolution over this range. Since the OCO‐2 satellite acquires 24 spectra per second and has much smaller ground‐pixels (higher spatial resolution), OCO‐2 has the potential to greatly advance SIF retrievals (Frankenberg et al., 2014).

While the Amazon is the largest overall terrestrial carbon sink (Hubau et al., 2020; Pan et al., 2011), the carbon fluxes in this region may vary significantly, or even change signs, during different seasons. For example, Jiang et al. (2021) showed that more CO_2_ is released to the atmosphere over the Amazon during the fire/dry season (August–October) than the wet season (January–March). To date, the biospheric contribution to such a seasonal increase in atmospheric CO_2_ is not well explored. Previous modeling studies suggested that there are reduced photosynthetic activities as a result of limited water during the dry season over the Amazon (e.g., Christoffersen et al., 2014; Werth & Avissar, 2004; Wu et al., 2017). However, observational studies (e.g., Bi et al., 2015; Guan et al., 2015; Huete et al., 2006; Morton et al., 2014; Restrepo‐Coupe et al., 2017; Saleska et al., 2016) demonstrated controversial results for photosynthetic activities during the dry season over the Amazon. Some studies (e.g., Bi et al., 2015; Huete et al., 2006; Saleska et al., 2016) suggested more photosynthetic activities, while others (e.g., Guan et al., 2015; Restrepo‐Coupe et al., 2017) suggested less photosynthetic activities during the dry season. In this article, we utilize SIF and column CO_2_ data from OCO‐2, precipitation data from the Global Precipitation Climatology Project (GPCP), and burned area data from the Moderate Resolution Imaging Spectrometer (MODIS) to investigate the interaction between the biosphere and the atmosphere during the Amazon fire/dry season. We also compare these results with those during the wet season.

## 2 Data

### 2.1 SIF Retrievals From OCO‐2

OCO‐2 was launched in July 2014 and has been providing CO_2_ and SIF data since September 2014 (Crisp et al., 2017). OCO‐2 consists of three grating spectrometers (Crisp et al., 2017). The fluorescence signal is measured at the Fraunhofer lines in the range 660–800 nm (Frankenberg et al., 2011; Sun et al., 2017). A combination of singular‐value decomposition and least‐square analysis were used to fit the fluorescence spectrum and estimate SIF (Frankenberg et al., 2011, 2014). The SIF data are regridded to 2° × 2° in latitude × longitude.

### 2.2 Column CO_2_ Retrievals From OCO‐2

High‐resolution spectra of reflected sunlight in the near‐infrared CO_2_ bands (1.61 and 2.06 μm) and the O_2_‐A band (0.76 μm) are utilized to retrieve the column‐averaged CO_2_, X_CO2_, from OCO‐2 (Crisp et al., 2004, 2017; Kuang et al., 2002). The difference between OCO‐2 column CO_2_ and Total Carbon Column Observing Network ground‐based Fourier Transform Spectrometer measurements is about 0.5 ppm (Wunch et al., 2017). OCO‐2 column CO_2_ data are available from September 2014 to the present and are regridded to 2° × 2° in latitude × longitude.

### 2.3 Precipitation Data From GPCP

Monthly mean GPCP Version 2.3 precipitation data (Adler et al., 2018; Huffman et al., 2012) are used in this article to estimate available water for the plants. Rain gauge data and precipitation data from different instruments are incorporated in the GPCP precipitation data (Adler et al., 2018; Kao et al., 2018). GPCP data are available from January 1979 to the present with a horizontal spatial resolution of 2.5° × 2.5° in latitude × longitude.

### 2.4 Burned Area Data From MODIS

Monthly mean MODIS burned area data (Giglio et al., 2018) are used to explore burned areas in the Amazon in this article. MODIS burned area data are available at 0.25° × 0.25° (latitude × longitude) from November 2000 to December 2019.

## 3 Results

### 3.1 Temporal Variability of SIF, Precipitation, and CO_2_ Over the Amazon Region

**Figure 1:**
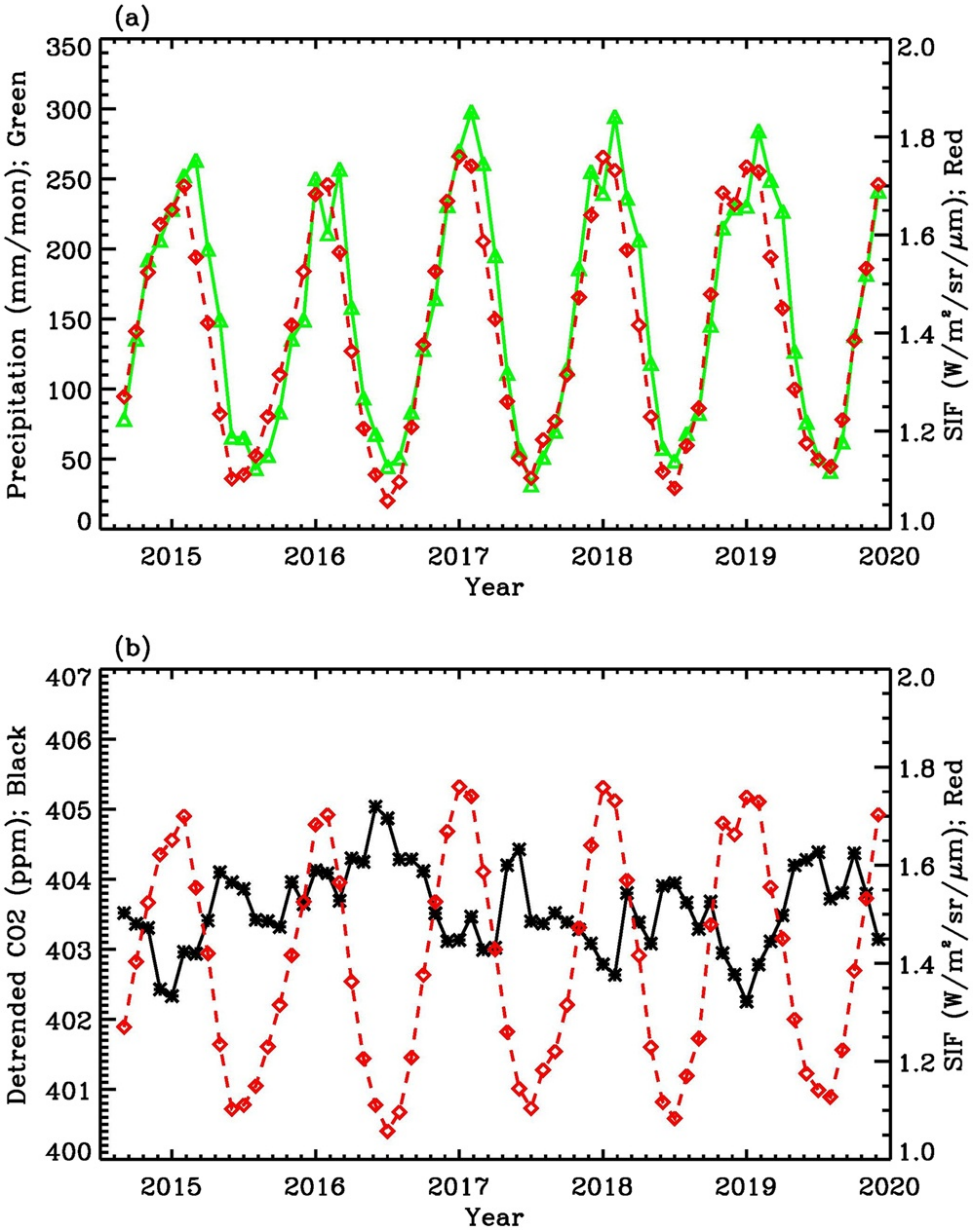
(a) Time series of GPCP precipitation (green line) and OCO‐2 SIF (red line) averaged over the Amazon basin. (b) Time series of detrended OCO‐2 CO_2_ (black line) and OCO‐2 SIF (red line) averaged over the Amazon basin. Units for precipitation, SIF, and CO_2_ are mm/mon, W/m^2^/sr/μm, and ppm, respectively.

**Figure 2:**
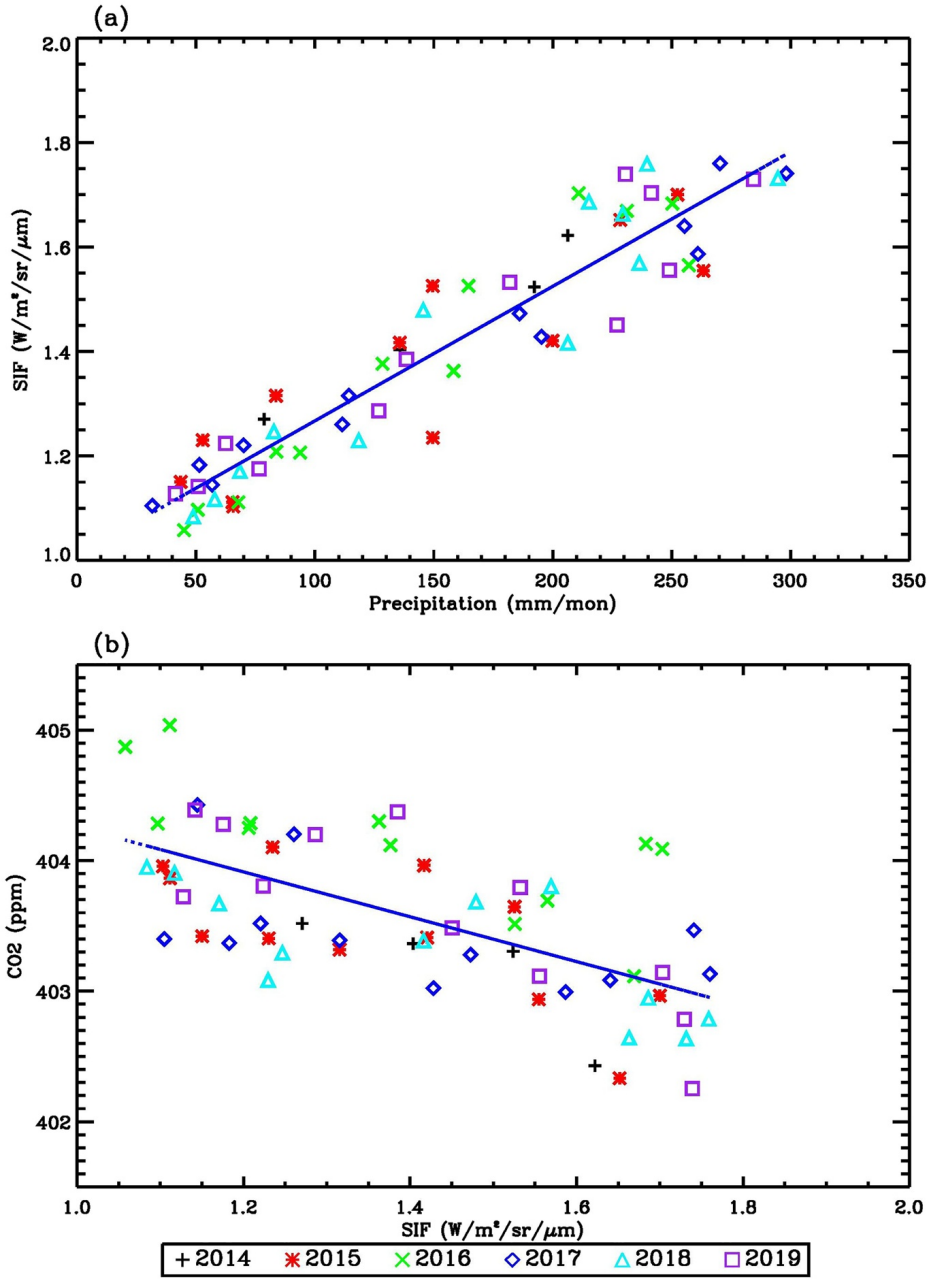
(a) Scatter plot of OCO‐2 SIF and GPCP precipitation. (b) Scatter plot of OCO‐2 CO_2_ and OCO‐2 SIF. Units for precipitation, SIF, and CO_2_ are mm/mon, W/m^2^/sr/μm, and ppm, respectively.

To explore the seasonal variations of SIF, precipitation, and CO_2_, we calculated the monthly mean value of OCO‐2 SIF, GPCP precipitation, and detrended OCO‐2 CO_2_ over the Amazon region from September 2014 to December 2019. The long‐term increasing trend of OCO‐2 CO_2_ is primarily due to anthropogenic emissions. Since we are not interested in the long‐term trend of CO_2_ in this article, we removed the linear trend of CO_2_ at each grid point (Bevington & Robinson, 2003; Jiang et al., 2021) before examining the temporal correlation between OCO‐2 SIF and CO_2_. OCO‐2 SIF (red line) and GPCP precipitation (green line) averaged over the Amazon region are shown in Figure 1a. High and low values of precipitation are related to high and low values of SIF, respectively, consistent with the fact that precipitation enhances photosynthesis. Low SIF values during the dry season over the Amazon are consistent with results in Wu et al. (2017), in which they suggested that there is less photosynthesis during the dry season than the wet season when water is limited. The correlation coefficient between OCO‐2 SIF and GPCP precipitation is 0.94, with a significance level of 1% that is estimated using the Monte Carlo method described in Jiang et al. (2004). A distribution of correlations was estimated from 3,000 correlation coefficients between the isospectral surrogate time series and the relevant indices. The distribution was then transformed into a normal distribution by the Fisher transformation (Devore, 1982). The significance level of the actual correlation within the normal distribution was determined (Jiang et al., 2004). A small value of the significance level refers to a high statistical significance. The slope for the linear regression of OCO‐2 SIF against precipitation (Figure 2a) is 2.57 × 10^−3^ W m^−2^ sr^−1^ μm^−1^ mm^−1^. The *R* ^2^ coefficient of determination is 0.9, which is close to 1.0, indicating that the fitted regression line describes the data very well.

Detrended OCO‐2 CO_2_ (black curve) and OCO‐2 SIF (red curve) averaged over the Amazon region are shown in Figure 1b. Since higher values of SIF imply higher photosynthetic activity, during which CO_2_ is removed from the atmosphere during the growth of plants, atmospheric CO_2_ values are expected to be lower over high SIF regions. The detrended OCO‐2 CO_2_ time series is anti‐correlated with OCO‐2 SIF, with a correlation coefficient of −0.64 at a significance level of 1%. The smaller correlation coefficient between CO_2_ and SIF compared to SIF and precipitation implies that atmospheric CO_2_ is also influenced by other factors (e.g., biomass burning, fossil fuel emissions, and circulation) that do not directly influence SIF, whereas SIF and precipitation are more directly correlated through plant photosynthesis. The slope for the linear regression of CO_2_ against SIF is −1.72 ppm (W m^−2^ sr^−1^ μm^−1^)^−1^. The *R* ^2^ coefficient of determination is 0.4, which suggests that the data are scattered and there is no simple relationship between CO_2_ and SIF. In addition to the annual cycle, there are also signals from the semi‐annual cycle (6‐month cycle) in SIF, precipitation, and CO_2_, as presented in Figure 1. The semi‐annual signal in SIF is related to the precipitation, while the semi‐annual signal in CO_2_ is related to the combination of photosynthesis and respiration (Jiang et al., 2012).

We explored the impact of 2015–2016 El Niño events on precipitation, SIF, and CO_2_ by removing the annual cycles from precipitation, SIF, and CO_2_. Annual cycles were estimated by averaging data in each month. These deseasonalized time series are shown in Figure S1 in Supporting Information S1. Previous studies (e.g., Liu et al., 2017) suggested that 2015–2016 El Niño events peaked in late 2015 with severe droughts. As shown in Figure S1a in Supporting Information S1, there are negative precipitation anomalies during November–December 2015, which led to negative anomalies of SIF during the same period. Low photosynthetic activity (low SIF) contributed to high atmospheric CO_2_ concentrations in November–December 2015 (Figure S1b in Supporting Information S1).

### 3.2 Seasonal Averages and Spatial Variability

**Figure 3:**
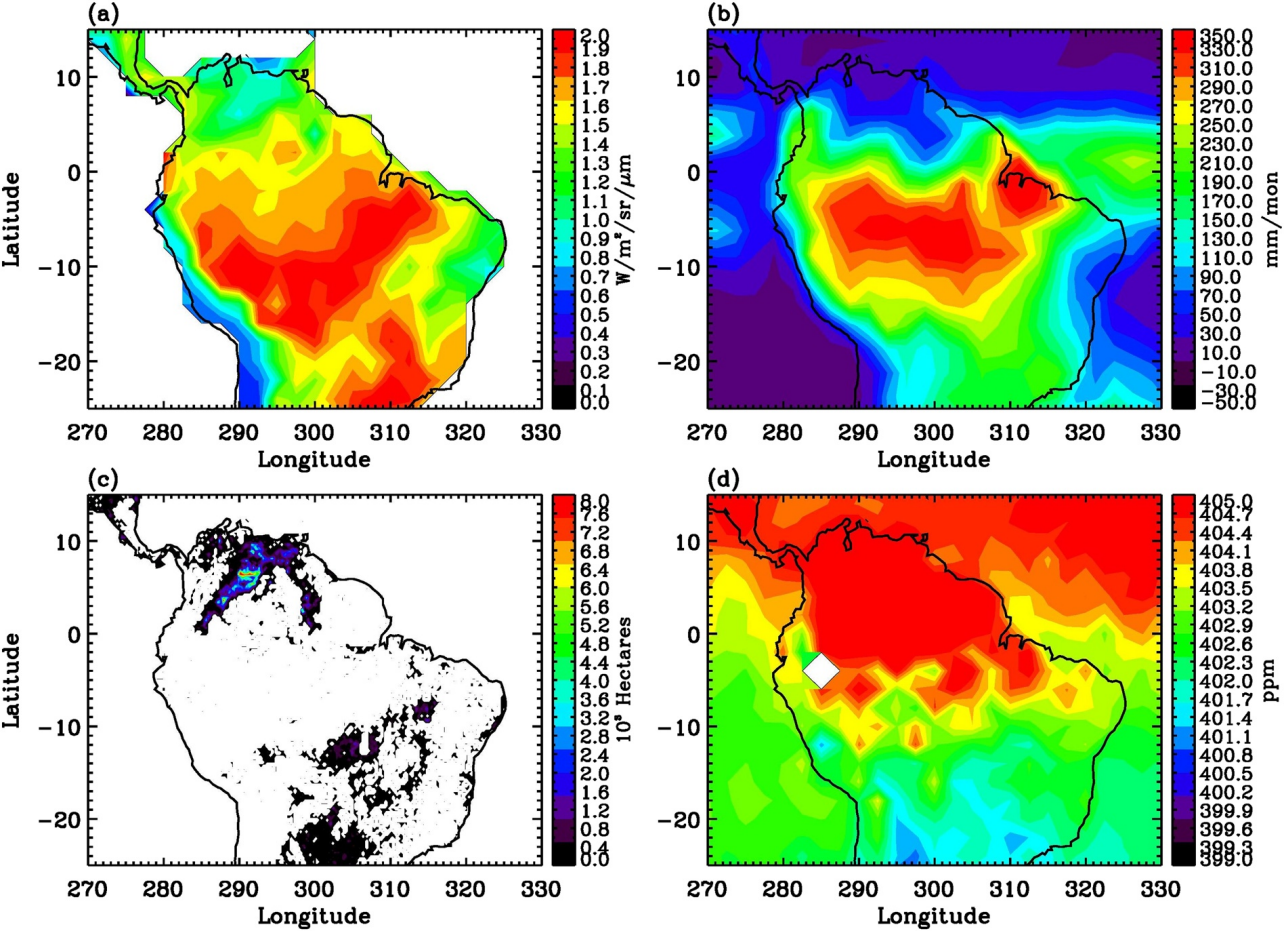
(a) OCO‐2 SIF averaged for the wet season. Units are W/m^2^/sr/μm. (b) GPCP precipitation averaged for the wet season. Units are mm/mon. (c) MODIS burned area averaged for the wet season. Units are 10^3^ Hectares. (d) OCO‐2 detrended CO_2_ averaged for the wet season. Units are ppm. Wet season refers to January–March 2015–2019.

**Figure 4:**
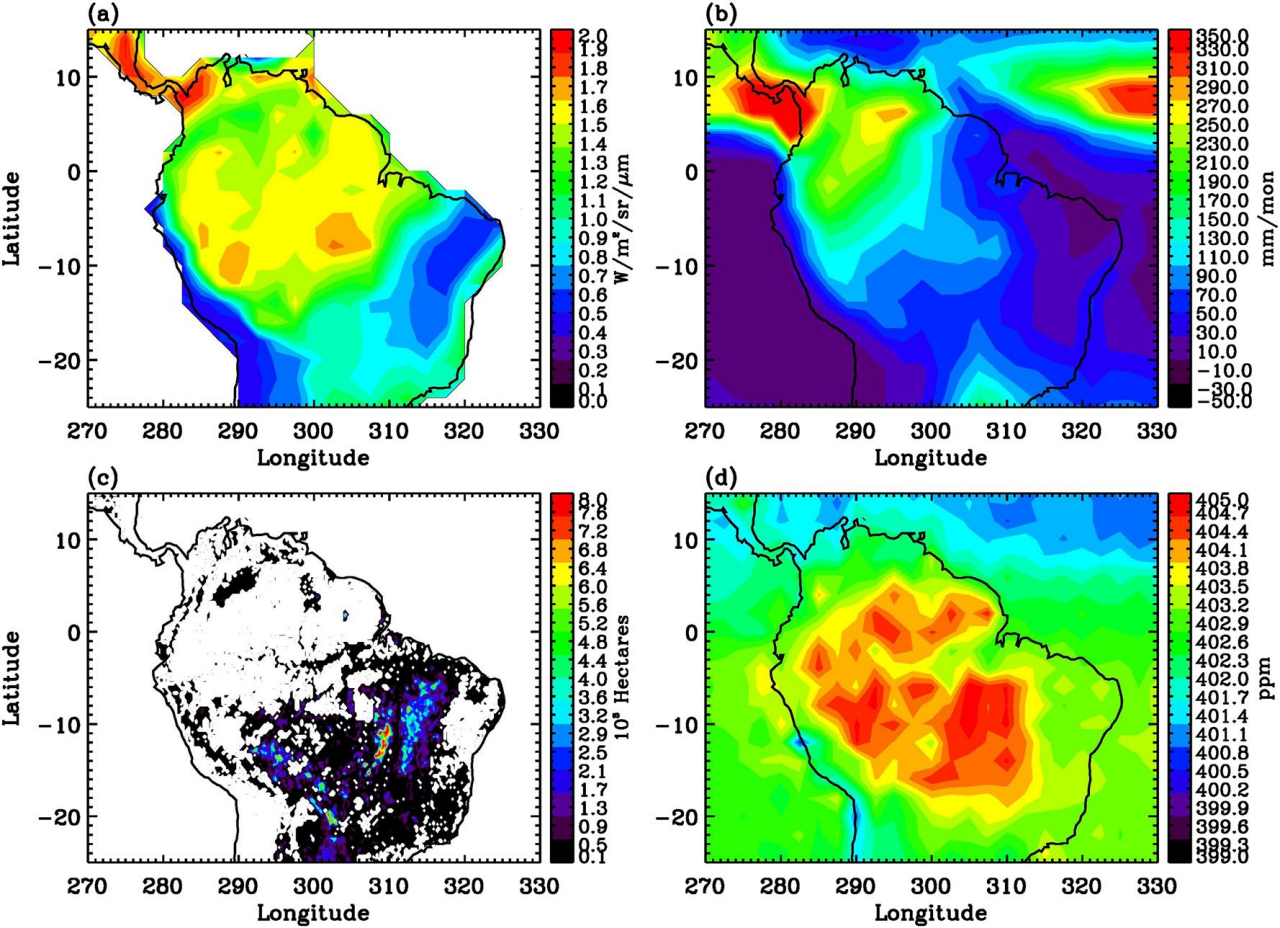
(a) OCO‐2 SIF averaged for the dry/fire season. Units are W/m^2^/sr/μm. (b) GPCP precipitation averaged for the dry/fire season. Units are mm/mon. (c) MODIS burned area averaged for the dry/fire season. Units are 10^3^ Hectares. (d) OCO‐2 detrended CO_2_ averaged for the dry/fire season. Units are ppm. Dry/fire season refers to August–October 2015–2019.

§3.1 explored the monthly variations averaged over the Amazon. In this section, we explore the spatial patterns in two major seasons in the Amazon region. In particular, we compare the spatial patterns of OCO‐2 SIF, GPCP precipitation, MODIS burned area, and OCO‐2 CO_2_ during January–March (Figure 3) and August–October (Figure 4) over the Amazon, to assess the photosynthetic activity of the region in the wet season and dry/fire season, respectively.

January–March is generally considered as the wet season in the Amazon, during which the region of high precipitation is the central and southern Amazon (Figure 3b) and fires dominate the northern region of South America, as indicated by the MODIS burned area in Figure 3c. Figures 3a, 3b, and 3d show that high‐SIF regions are co‐located with high‐precipitation and low‐CO_2_ regions in the central and southern regions. In contrast, low‐SIF regions are co‐located with more burned areas and low precipitation in the northern and eastern parts of South America.

August–October is generally considered the dry/fire season in the Amazon, with fires mostly found in the southern Amazon, as indicated by the MODIS burned area in Figure 4c. Figure 4a shows that SIF values are higher over the northern region of the South America and lower over the southern and eastern regions of the South America. The high‐SIF regions coincide with the high‐precipitation regions shown in Figure 4b, again consistent with the fact that precipitation enhances photosynthetic activities. In contrast, the low SIF region in the southern America during this season is likely due to the fires that have reduced the vegetation in the area. The high CO_2_ values over the southern and central regions of the Amazon are a result of the fires (Jiang et al., 2021).

**Figure 5:**
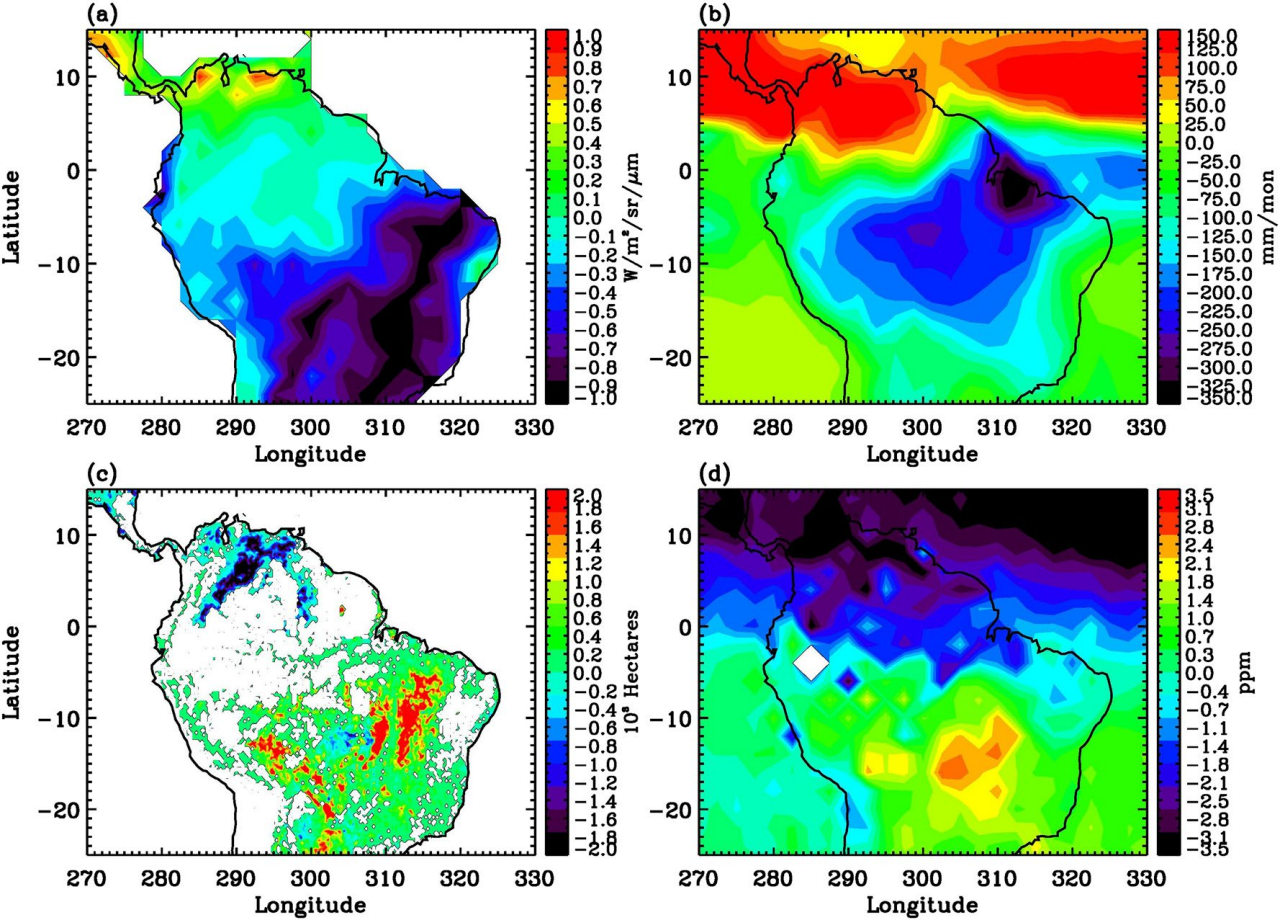
(a) OCO‐2 SIF difference between the dry season and the wet season. Units are W/m^2^/sr/μm. (b) GPCP precipitation difference between the dry season and the wet season. Units are mm/mon. (c) MODIS burned area difference between the dry season and the wet season. Units are 10^3^ Hectares. (d) OCO‐2 CO_2_ difference between the dry/fire season and the wet season. Units are ppm.

The relationships among SIF, precipitation, burned area, and CO_2_ can be seen more easily in the differences of these variables between the dry/fire season and the wet season. Figure 5a shows that the SIF differences are positive over the northern Amazon and negative over the central and southern regions of the Amazon, which are consistent with the precipitation differences shown in Figure 5b. Low SIF values over the southern Amazon are also consistent with more burned areas during the dry/fire season. High SIF values contribute to low CO_2_ over the northern Amazon and low SIF values contribute to high CO_2_ over the southern Amazon.

The relationship between the SIF difference (dry season‐wet season) and the precipitation difference (dry season‐wet season) over 270°E−330°E, 25°S–15°S is shown in Figure S2a in Supporting Information S1. There is a positive correlation between OCO‐2 SIF difference and precipitation difference. The SIF difference (dry season‐wet season) is positive when there is more precipitation, implying there is more photosynthesis when more water is available. Over the southern part of the Amazon region, there are larger negative precipitation anomalies in the dry/fire season than the wet season, which will lead to negative SIF anomalies. A scatter‐plot for OCO‐2 SIF difference and OCO‐2 CO_2_ difference is shown in Figure S2b in Supporting Information S1. There is a negative correlation between OCO‐2 SIF differences and OCO‐2 CO_2_ differences. Over the southern Amazon region, negative SIF anomalies (less photosynthetic activity) contribute to positive atmospheric CO_2_ anomalies.

In addition to precipitation and burned areas, we also investigated differences between the vapor pressure deficit (VPD) during the wet and dry/fire seasons. VPD is defined as the difference between the saturation vapor pressure and the actual vapor pressure (Barkhordarian et al., 2019). We calculated VPD for the two seasons using surface air temperature and relative humidity from NCEP2 reanalysis data sets (Kanamitsu et al., 2002). Results are shown in Figure S3 in Supporting Information S1. As shown in Figure S3a in Supporting Information S1, the VPD values are low over the central and southern regions of the Amazon during the wet season. Low VPD values suggest that the air is close to saturation and the open stomata of the plants will remove CO_2_ from the atmosphere and facilitate photosynthesis (high SIF values in Figure 3a). During the dry/fire season, the VPD is high over the eastern region of the Amazon (Figure S3b in Supporting Information S1). As a result of high VPD values, the stomata will partially close to retaining moisture (Lange et al., 1971), which will limit the uptake of CO_2_ and suppress photosynthetic activity over the eastern region of the Amazon (Figure 4a). The difference in VPD values between the dry/fire season and the wet season is shown in Figure S3c in Supporting Information S1. The VPD differences are positive over the southeastern region of the Amazon, consistent with negative SIF differences over this same region, shown in Figure 5a. Photosynthetically active radiation (PAR) data (Gelaro et al., 2017) were also analyzed for the wet season and dry/fire season. Results are shown in Figure S4 in Supporting Information S1. PAR is defined as the solar radiation between 400 and 700 nm, which is involved in photosynthetic processes (McCree, 1972). The amount of PAR can be influenced by factors such as location, season, and cloud cover. As shown in Figure S4a in Supporting Information S1, the PAR values are low over the Amazon during the wet season, as a result of high fractions of cloud coverage. During the dry/fire season, PAR is higher than during the wet season (Figure S4b in Supporting Information S1). The difference in PAR values between the dry/fire season and the wet season is shown in Figure S4c in Supporting Information S1. The PAR differences are positive over the Amazon. Positive anomalies of PAR can contribute to positive SIF anomalies over the northern part of Amazon. Over the southern Amazon, the negative SIF anomalies are related to the limited water (Figure 5b) and high VPD values (Figure S3c in Supporting Information S1).

**Figure 6:**
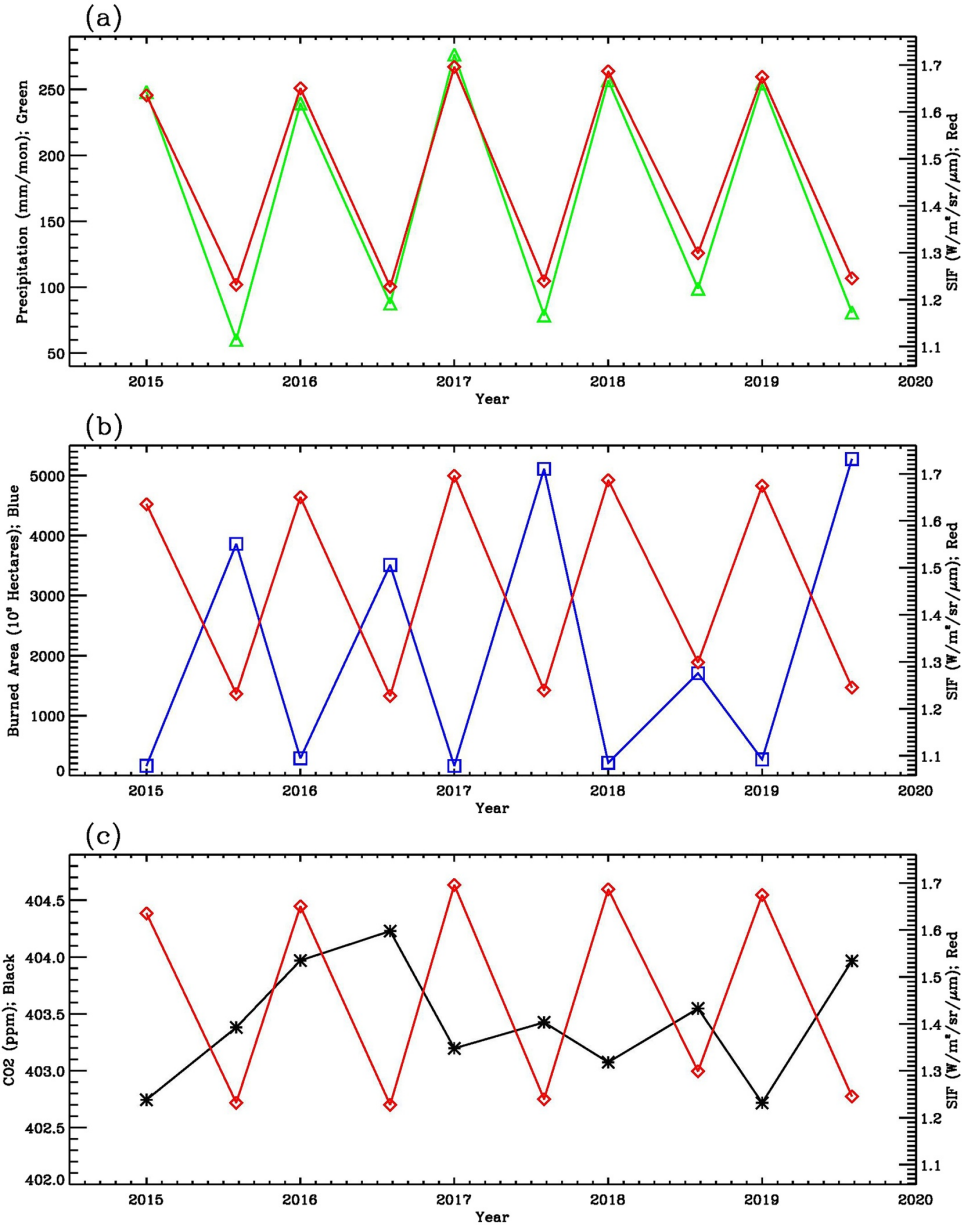
(a) Time series of OCO‐2 SIF (red line) and GPCP precipitation (green line) averaged over the Amazon basin for wet seasons (January–March) and dry/fire seasons (August–October). (b) Time series of OCO‐2 SIF (red line) and MODIS burned area (blue line) averaged over the same region as (a). (c) Time series of OCO‐2 SIF (red line) and OCO‐2 CO_2_ (black line) averaged over the same region as (a). Units for precipitation, SIF, burned area, and CO_2_ are mm/mon, W/m^2^/sr/μm, 10^3^ Hectares, and ppm, respectively.

To explore the temporal variations of OCO‐2 SIF, GPCP precipitation, MODIS burned area, and OCO‐2 CO_2_, we averaged these variables in the dry/fire and wet seasons, respectively. Figure 6a shows that SIF and precipitation time series follow each other closely, with a correlation coefficient of 0.99 (6% significance level); a lower significance level, relative to those for the monthly times series (Figure 1), is obtained because there are fewer data points in the seasonal time series. Figure 6b shows that SIF and burned area seasonal time series are anti‐correlated, with a correlation coefficient −0.92 (7% significance level). As shown in Figure 6c, there is an anti‐correlation between SIF and CO_2_ with a correlation coefficient of −0.60 (8% significance level). There is an exception in the wet season of 2016, when SIF was anomalously high, but CO_2_ did not decrease, as would have been expected based on the wet seasons of other years. This exceptional case may be explained by the increase in soil and plant respiration as a response to the El Niño event that year (Chatterjee et al., 2017; Jiang et al., 2021; Levine et al., 2019; Liu et al., 2017).

## 4 Conclusions

Temporal and spatial variations of OCO‐2 SIF, GPCP precipitation, MODIS burned area, and OCO‐2 CO_2_ were investigated over the Amazon region. As revealed in the time series of regionally averaged SIF and precipitation, there is a positive correlation (*R* = 0.94) between these two parameters, consistent with the fact that more precipitation leads to higher photosynthetic activity and hence higher values of SIF, which in turn enhances the terrestrial uptake of CO_2_ and thereby reduces the atmospheric CO_2_ above the region, as indicated by a negative correlation (*R* = −0.64) between SIF and CO_2_.

This spatial pattern shifts as the seasons change. During the wet season, precipitation values are high and VPD values are low over the central and southern regions of the Amazon. Associated with high precipitation and low VPD, SIF values are high over the central and southern regions of the Amazon. High SIF values indicate enhanced photosynthesis, which uptakes CO_2_ and results in low CO_2_ concentration in the atmosphere. During the dry/fire season, SIF values are low over the southern and eastern regions of the Amazon as a result of low precipitation, high VPD, and more burned area in these regions. As a result of the fires and low photosynthetic drawdown, CO_2_ values are high over these regions during the dry/fire season.

Temporal variations of SIF, precipitation, CO_2_, and burned area during wet and dry seasons were also investigated. There is a positive correlation (*R* = 0.99) between SIF and precipitation, a negative correlation (*R* = −0.92) between SIF and burned area, and a negative correlation (*R* = −0.60) between SIF and CO_2_. During the wet season, burned area is low and precipitation and SIF values are high, resulting in low CO_2_ values. During the dry/fire season, burned area is large and precipitation and SIF values are low, resulting in high CO_2_ values.

In summary, we have found that the Amazon rainforest, the largest biospheric carbon sink, switches to a carbon source during the dry/fire season. There is more atmospheric CO_2_ over the Amazon region during the dry/fire season than the wet season as a result of enhanced biomass burning (Figure 5c) and reduced photosynthetic activities (Figure 5a) during the dry/fire season. Previous observational studies demonstrated controversial results for photosynthetic activity during the dry season over the Amazon (Bi et al., 2015; Guan et al., 2015; Huete et al., 2006; Morton et al., 2014; Restrepo‐Coupe et al., 2017; Saleska et al., 2016). To better elucidate the contribution from the biosphere to the atmosphere, we have explored photosynthetic activity using OCO‐2 SIF data over the Amazon region during the dry/fire season. We found that photosynthetic activity is low during the dry/fire season, especially over the southern Amazon region, as a result of low precipitation and high VPD. Low photosynthetic activity (low SIF) contributes to high atmospheric CO_2_ over the Amazon region during the dry/fire season. Reduced photosynthetic activity (low SIF) and enhanced atmospheric CO_2_ concentrations were also found over the Amazon region during the 2015–2016 El Niño events. Results from this study can help us better understand the impact of photosynthesis on atmospheric CO_2_ during the dry/fire season. Since it is still a challenge to simulate the variability of CO_2_ using chemistry‐transport models, results obtained from this study can be used to constrain/improve such numerical models in the future, especially the contribution from the biosphere.

## Supporting information

Supporting Information S1

## Data Availability

GPCP Version 2.3 precipitation data can be downloaded at https://psl.noaa.gov/data/gridded/data.gpcp.html. OCO‐2 Version 10 SIF and column CO_2_ data can be downloaded at https://co2.jpl.nasa.gov/#mission=OCO-2. MODIS burned area data can be downloaded at http://modis-fire.umd.edu/ (Please see Section 4.1 of the User's Manual for details ‐ https://modis-fire.umd.edu/files/MODIS_C6_BA_User_Guide_1.3.pdf).
