# Supplementary material for: Seasonal Variations of Solar‐Induced Fluorescence, Precipitation, and Carbon Dioxide Over the Amazon
Source: Earth Space Sci. 2022 Jan 18;9(1):e2021EA002078. doi: 10.1029/2021EA002078 (PMC9285695; doi:10.1029/2021EA002078)

# Supplementary Material for Seasonal Variations of Solar-Induced Fluorescence, Precipitation, and Carbon Dioxide Over the Amazon

Ronald Albright^1^, Abigail Corbett^1,2^, Xun Jiang^1*^, Ellen Creecy^1^, Sally Newman^3^, King-Fai Li^4^, Mao-Chang Liang^5^, Yuk L. Yung^6,7*^

1 Department of Earth & Atmospheric Sciences, University of Houston, Houston, 77004 TX, USA

2 SeekOps Inc, Austin, 78730 TX, USA

3 Bay Area Air Quality Management District, San Francisco, 94105 CA, USA

4 Department of Environmental Sciences, University of California, 900 University Avenue, Riverside, 92521 CA, USA

5 Institute of Earth Sciences, Academia Sinica, 11529 Taipei, Taiwan

6 Division of Geological and Planetary Sciences, California Institute of Technology, 1200 East California Boulevard, Pasadena, 91125 CA, USA

7 Jet Propulsion Laboratory, 4800 Oak Grove Dr., Pasadena, 91109 CA, USA

* Correspondence: xjiang7@uh.edu

To explore the impact of 2015-2016 El Niño events on precipitation, Solar-Induced Fluorescence (SIF), and CO_2_, we removed the annual cycles from precipitation, SIF, and CO_2_. Annual cycles were estimated by averaging data in each month. Deseasonalized timeseries of precipitation, SIF, and CO_2_ are shown in Figure S1. Previous studies (e.g., Liu et al., 2017) suggested that 2015-2016 El Niño events peaked in the late 2015 with severe droughts. As shown in Figure S1a, there are negative precipitation anomalies during late 2015, which correspond to the peak El Niño months. Low precipitation led to negative anomalies of SIF during Nov-Dec 2015. Low photosynthetic activity (low SIF) contributed to high atmospheric CO_2_ concentration in Nov-Dec 2015 (Figure S1b).

The relationship between SIF differences (ASO-JFM) and precipitation differences (ASO-JFM) over 270°E-330°E, 25°S-15°N is shown in Figure S2a. There is a positive correlation between OCO-2 SIF difference and precipitation difference. SIF difference (ASO-JFM) is positive when there is more precipitation, implying there is more photosynthesis when more water is available. Over the southern Amazon region (red diamonds in Figure S2a), there are larger negative precipitation anomalies in the dry/fire season than in the wet season, which lead to negative SIF anomalies. A scatterplot is shown for OCO-2 SIF difference and OCO-2 CO_2_ difference in Figure S2b. There is a negative correlation between OCO-2 SIF difference (ASO-JFM) and OCO-2 CO_2_ difference (ASO-JFM). Over the southern Amazon region, negative SIF anomalies (less photosynthetic activity) contribute to positive atmospheric CO_2_ anomalies.

Using surface air temperature and relative humidity from NCEP2 Reanalysis data sets (Kanamitsu et al., 2002), we calculated the vapor pressure deficit (VPD) for the wet season (Jan-Mar, 2015-2019) and the dry season (Aug-Oct, 2015-2019) over the Amazon, respectively. VPD is estimated as the difference between the saturation vapor pressure and actual vapor pressure (based on Equation 1 in Barkhordarian et al., 2019). High VPD values suggest that the air is more undersaturated in water vapor, while low VPD values suggest air is close to saturation.

As shown in Figure S3a, the VPD values are very low over the central and southern regions of the Amazon during the wet season (Jan-Mar, 2015-2019). Low VPD values suggest that the air is close to saturation and the open stomata on leaves will remove CO_2_ from the atmosphere and facilitate photosynthesis, which is consistent with high SIF values over the central and southern regions of the Amazon shown in Figure 3a. During the dry/fire season, the VPD is high over the eastern region of the Amazon (Figure S3b). As a result of high VPD values, the stomata on leaves will partially close to retain moisture for the plants (Lange et al., 1971), which will limit the uptake of CO_2_ and suppress photosynthesis over the eastern region of the Amazon as shown in Figure 4a. The difference in VPD values between the dry/fire season and the wet season is shown in Figure S3c. The VPD differences are positive over the southeastern regions of the Amazon, which are consistent with negative SIF differences over southeastern regions of the Amazon shown in Figure 5a.

We also calculated the photosynthetically active radiation (PAR) for the wet season (Jan-Mar, 2015-2019) and the dry season (Aug-Oct, 2015-2019) over the Amazon, respectively. PAR is defined as the solar radiation between 400 and 700 nm, which is involved in photosynthetic processes (McCree, 1972). The amount of PAR can be influenced by factors such as location, season, and cloud cover. Monthly mean PAR data from The Second Modern-Era Retrospective analysis for Research and Applications (MERRA-2) (Gelaro et al., 2017) was used in this analysis. As shown in Figure S4a, the PAR values are very low over the Amazon as a result of the high fraction of cloud coverage during the wet season (Jan-Mar, 2015-2019). During the dry/fire season, PAR is high over the Amazon (Figure S4b). The difference in PAR values between the dry/fire season and the wet season is shown in Figure S4c. The PAR differences are positive over the Amazon. Positive anomalies of PAR can contribute to positive SIF anomalies over the northern part of Amazon. Over the southern Amazon, the negative SIF anomalies are more constrained by the limited water (Figure 5b) and high VPD values (Figure S3c).

**References**

Barkhordarian, A., Saatchi, S. S., Behrangi, A., Loikith, P. C., & Mechoso, C. R. (2019). A recent systematic increase in vapor pressure deficit over tropical south America. *Scientific Reports, 9*, 15331. https://doi.org/10.1038/s41598-019-51857-8

Kanamitsu, M., Ebisuzaki, W., Woollen, J., Yang, S.-K., Hnilo, J. J., Fiorino, M., & Potter, G. L. (2002). NCEP-DOE AMIP-II Reanalysis (R-2). *Bull. Am. Meteorol. Soc., 83*, 1631–1643. [https://doi.org/10.1175/BAMS-83-11-1631(2002)083<1631:NAR>2.3.CO;2](https://doi.org/10.1175/BAMS-83-11-1631(2002)083%3c1631:NAR%3e2.3.CO;2)

Gelaro, R., McCarty, W., Suarez, M. J., Todling, R., Molod, A., Takacs, L., et al. (2017). The Modern-Era Retrospective Analysis for Research and Applications, Version 2 (MERRA-2). *J. Climate*, *30*, 5419-5454. <https://doi.org/10.1175/JCLI-D-16-0758.1>.

Lange, O. L., Losch, R., Schulze E. D., & Kappen, L. (1971). Responses of stomata to changes in humidity. *Planta*, *100*, 76-86.

Liu, J.., Bowman, K. W., Schimel, D. S., Parazoo, N. C., Jiang, Z., Lee, M. et al. (2017). Contrasting carbon cycle responses of the tropical continents to the 2015-2016 El Nino. *Science*, *358*, eaam5690.

McCree, K. J. (1972). Test of current definitions of photosynthetically active radiation against leaf photosynthesis data. *Agricultural Meteorology*, *10*, 443-453. https://doi.org/10.1016/0002-1571(72)90045-3.

Fig. S1: Time series of deseasonalized GPCP precipitation (green line) and deseasonalized OCO-2 SIF (red line) averaged over the Amazon basin. (b) Time series of deseasonalized and detrended OCO-2 CO_2_ (black line) and deseasonalized OCO-2 SIF (red line) averaged over the Amazon basin. Units for precipitation, SIF, and CO_2_ are mm/mon, W/m^2^/sr/μm, and ppm, respectively.


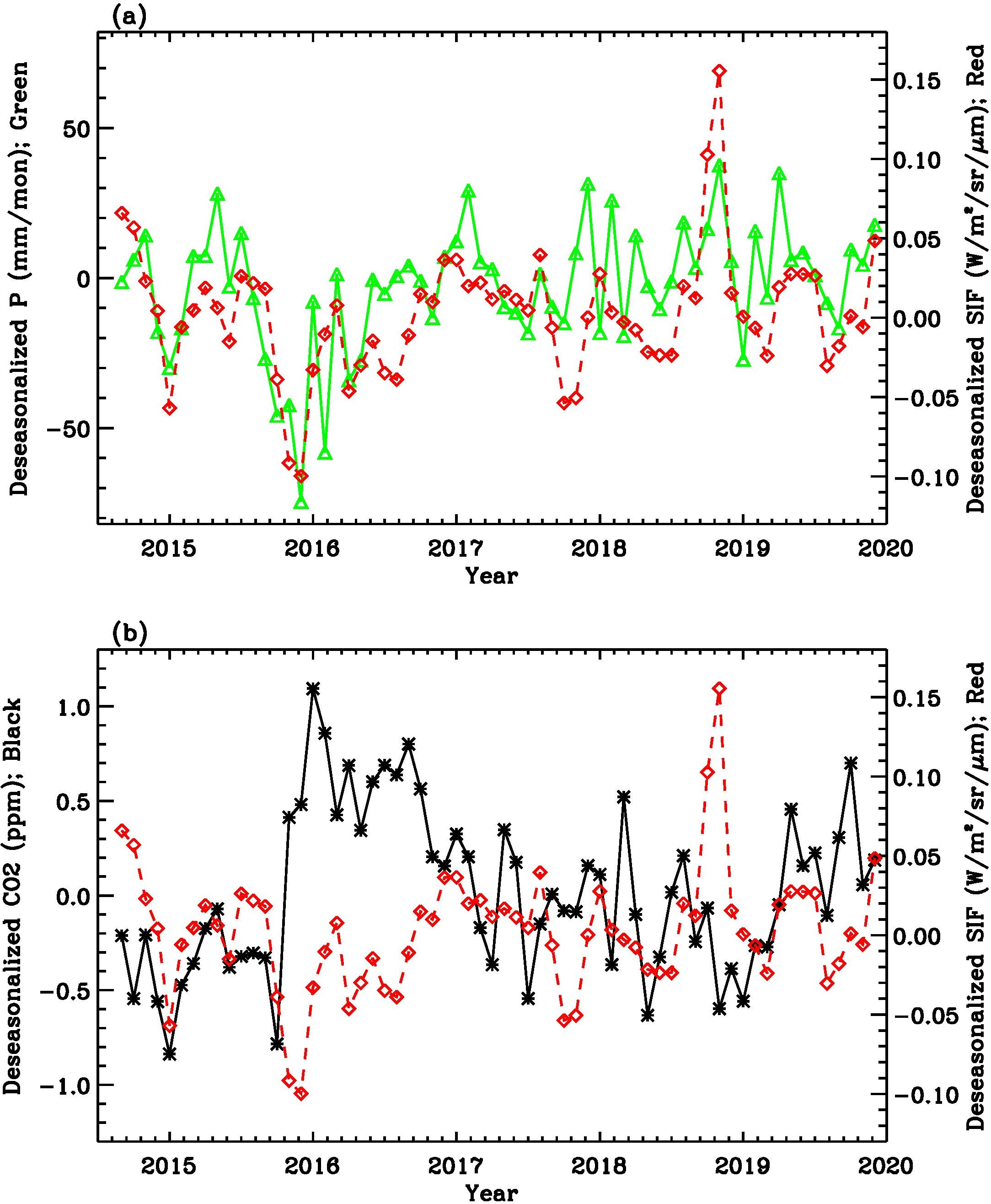


Fig. S2: (a) Scatter plot of OCO-2 SIF difference and precipitation difference between dry/fire season (Aug-Oct) and wet season (Jan-Mar) over 270°E-330°E, 25°S-15°N. (b) Scatter plot of OCO-2 SIF difference and OCO-2 column CO_2_ difference between dry/fire season (Aug-Oct) and wet season (Jan-Mar) over 270°E-330°E, 25°S-15°N. Blue lines are linear fits of the scatter plots. Red diamonds refer to data over the southern Amazon region. Blue diamonds refer to data in other region.


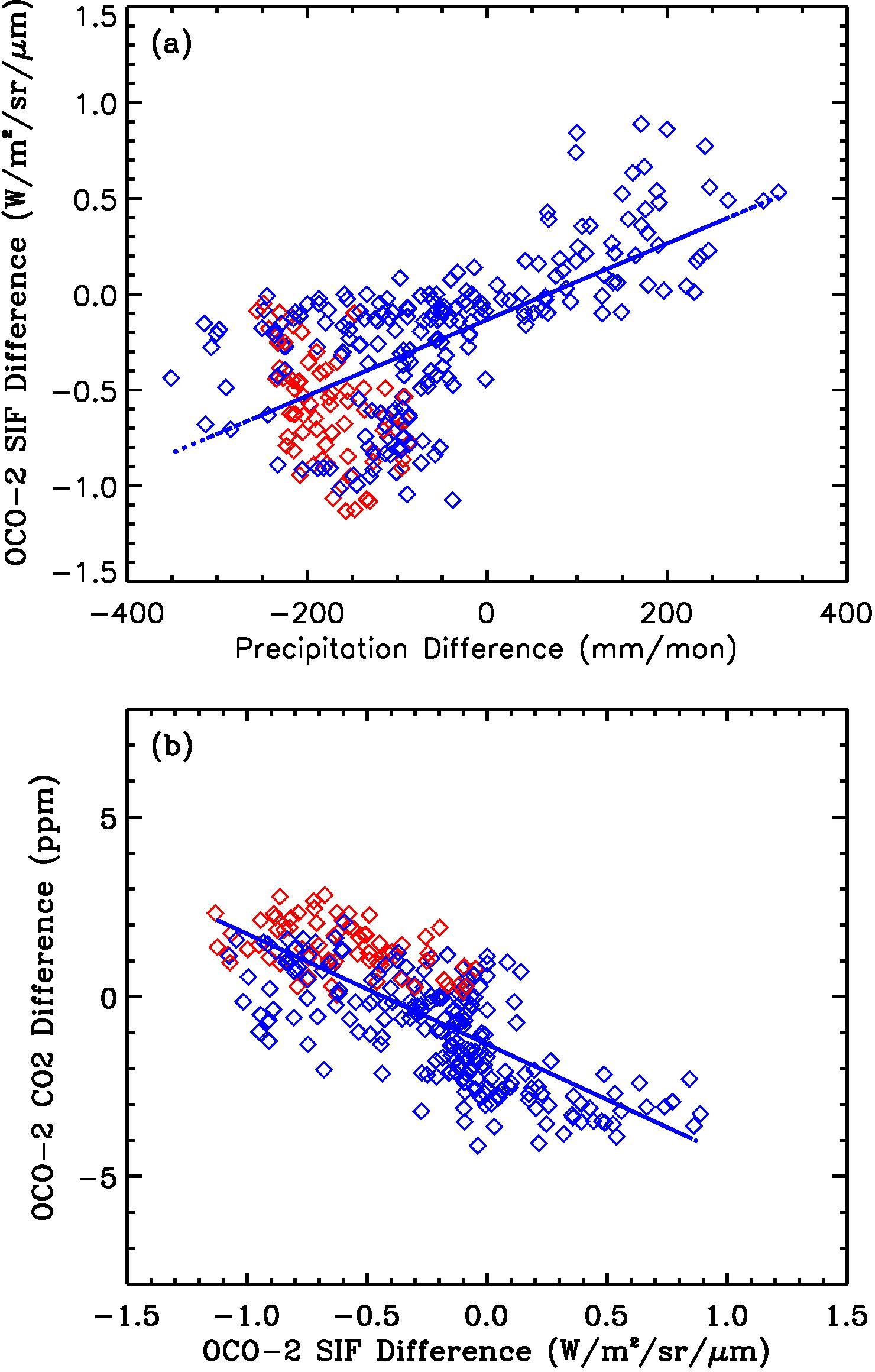


Fig. S3: (a) Vapor pressure deficit (VPD) averaged for the wet season (Jan-Mar, 2015-2019). (b) VPD averaged for the dry/fire season (Aug-Oct, 2015-2019). (c) VPD difference between the dry/fire season and the wet season. Units are hPa.


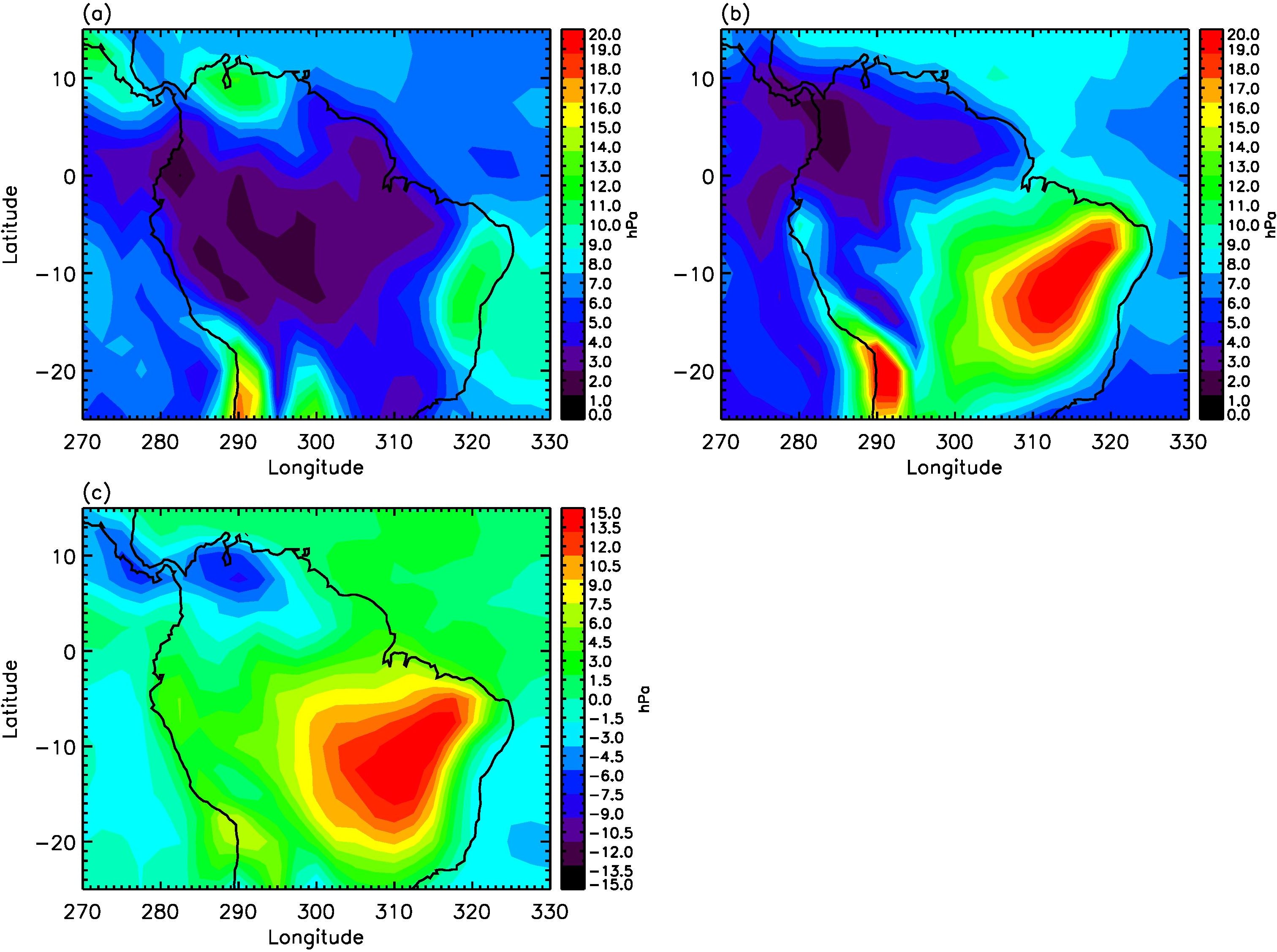


Fig. S4: (a) Photosynthetically Active Radiation (PAR) averaged for the wet season (Jan-Mar, 2015-2019). (b) PAR averaged for the dry/fire season (Aug-Oct, 2015-2019). (c) PAR difference between the dry/fire season and the wet season. Units are W/m^2^.


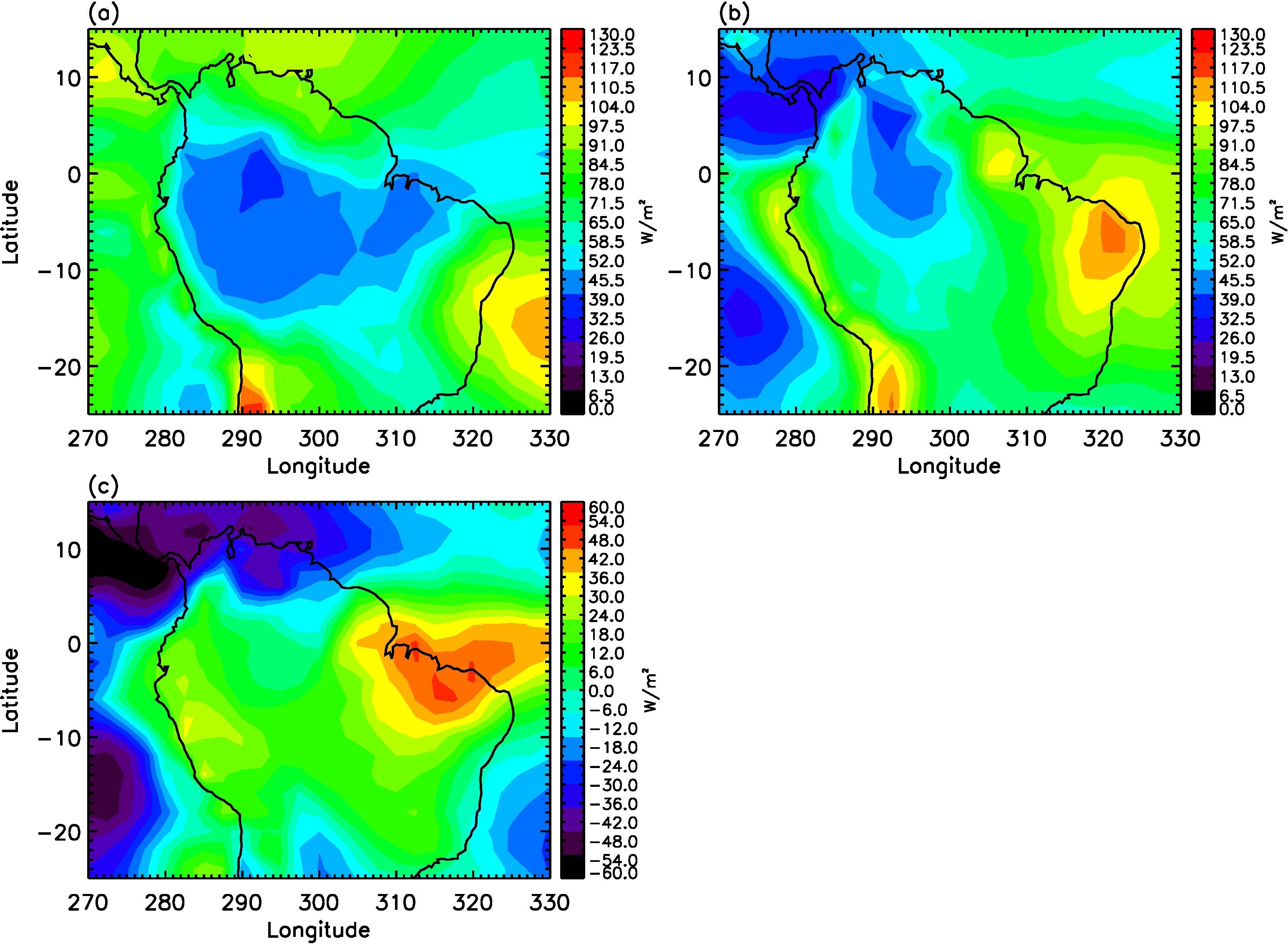

Supplement: Supplementary file 1 — Supporting Information S1 [file ESS2-9-0-s001.docx]
